# Comparison of characteristics, management and outcomes in hospital-onset and community-onset stroke: a multi-centre registry-based cohort study of acute stroke

**DOI:** 10.1007/s10072-022-06015-w

**Published:** 2022-03-23

**Authors:** David Fluck, Christopher H. Fry, Suzanne Rankin, Giosue Gulli, Brendan Affley, Jonathan Robin, Puneet Kakar, Pankaj Sharma, Thang S. Han

**Affiliations:** 1 Department of Cardiology, Ashford and St Peter’s NHS Foundation Trust, Chertsey, GU9 0PZ UK; 2 School of Physiology, Pharmacology and Neuroscience, University of Bristol, Bristol, BS8 1TD UK; 3 Department of Stroke, Ashford and St Peter’s NHS Foundation Trust, Chertsey, GU9 0PZ UK; 4 Department of Acute Medicine, Ashford and St Peter’s NHS Foundation Trust, Chertsey, GU9 0PZ UK; 5 Department of Stroke, Epsom and St Helier University Hospitals, Epsom, KT18 7EG UK; 6 Department of Clinical Neuroscience, Imperial College Healthcare NHS Trust, London, W6 8RF UK; 7 Institute of Cardiovascular Research, Royal Holloway University of London, Egham, TW20 0EX UK; 8 Department of Endocrinology, Ashford and St Peter’s NHS Foundation Trust, Chertsey, GU9 0PZ UK

**Keywords:** Healthcare economics, Mortality, Morbidity, Length of stay, Nosocomial infections, Multimorbidity

## Abstract

**Objective:**

Hospital-onset stroke (HOS) is associated with poorer outcomes than community-onset stroke (COS). Previous studies have variably documented patient characteristics and outcome measures; here, we compare in detail characteristics, management and outcomes of HOS and COS.

**Methods:**

A total of 1656 men (mean age ± SD = 73.1 years ± 13.2) and 1653 women (79.3 years ± 13.0), with data prospectively collected (2014–2016) from the Sentinel Stroke National Audit Programme, were admitted with acute stroke in four UK hyperacute stroke units (HASU). Associations between variables were examined by chi-squared tests and multivariable logistic regression (COS as reference).

**Results:**

There were 272 HOS and 3037 COS patients with mean ages of 80.2 years ± 12.5 and 76.4 years ± SD13.5 and equal sex distribution. Compared to COS, HOS had higher proportions ≥ 80 years (64.0% vs 46.4%), congestive heart failure (16.9% vs 4.9%), atrial fibrillation (25.0% vs 19.7%) and pre-stroke disability (9.6% vs 5.1%), and similar history of stroke, hypertension, diabetes, stroke type and severity of stroke. After age, sex and co-morbidities adjustments, HOS had greater risk of pneumonia: OR (95%CI) = 1.9 (1.3–2.6); malnutrition: OR = 2.2 (1.7–2.9); immediate thrombolysis complications: OR = 5.3 (1.5–18.2); length of stay on HASU > 3 weeks: OR = 2.5 (1.8–3.4); post-stroke disability: OR = 1.8 (1.4–2.4); and in-hospital mortality: OR = 1.8 (1.2–2.4), as well as greater support at discharge including palliative care: OR = 1.9 (1.3–2.8); nursing care: OR = 2.0 (1.3–4.0), help for daily living activities: OR = 1.6 (1.1–2.2); and joint-care planning: OR = 1.5 (1.1–1.9).

**Conclusions:**

This detailed analysis of underlying differences in subject characteristics between patients with HOS or COS and adverse consequences provides further insights into understanding poorer outcomes associated with HOS.

## Introduction

In high-income countries, hospital-onset stroke (HOS) accounts for 5–17% of all hospitalised acute stroke patients, whilst community-onset stroke (COS) accounts for the remainder [1–5]. The numbers of patients with HOS per annum have been estimated to be 35,000–75,000 in the USA [2] and 4000 in England and Wales [1]. Compared to patients with COS, those with HOS remain at greater risk of poor outcomes including post-stroke disability [4–6], mortality [5–7], prolonged length of stay (LOS) in hospital [8], and less likely to be discharged home [5, 6]. Despite its worse outcomes, HOS has often been overlooked in major reports [1, 9, 10]. Consequently, progress on management and outcomes over the years for this group of patients is little-known.

Multiple factors are likely to contribute to poor outcomes amongst patient with HOS, including their underlying health status, the management of acute stroke, such as prompt neuroimaging and diagnosis, and clinical and supportive treatment. Furthermore, most HOS cases occurred whilst on medical departments (63.4%) with the remainder on surgical departments (36.7%) [4]. In a study of US patients, up to 40% of HOS cases were admitted with a cardiovascular- or neurologically related condition, and 68% underwent an invasive diagnostic or surgical procedure before developing their acute stroke [5]. A similar observation was made amongst Korean patients, showing 46% of HOS occurred in cardiology or cardiovascular surgery departments, and 60% of HOS had undergone surgical procedures [11]. However, most studies partially documented these underlying factors but without a comprehensive report on underlying functional status, management and outcome measures, as well as supportive care during admission and at discharge.

In this study of patients with COS and HOS, recruited as part of the Sentinel Stroke National Audit Programme (SSNAP) [12], we have described in detail the characteristics of these patients. This includes: age; sex; co-morbidities most commonly associated with risk of stroke; pre-stroke disability and the severity of stroke; and management of stroke including swallow screening, thrombolysis and supportive care during admission. We have also described a range of clinical outcomes including: nosocomial infections; malnutrition; prolonged LOS; post-stroke disability; in-hospital mortality; as well as supportive care at discharge including palliation, nursing care, help for activities of daily living (ADL), joint care planning and weekly visits.

## Methods

### Study design, participants and setting

We analysed prospectively collected data from the UK national register of stroke care. These data contain clinical characteristics and care quality determinants of patients admitted to acute care hospitals in England and Wales [12]. Data for this study were gathered from 3309 patients consecutively admitted with an acute stroke to four UK hyperacute stroke units (HASU) in the south of England between January 2014 and February 2016 [13, 14].

### Socio-demographic factors and medical history

Socio-demographic details were collected and documented by stroke consultants and nurse specialists; including age at diagnosis, sex and co-morbidities: congestive heart failure (CHF), atrial fibrillation (AF), history of previous stroke, hypertension, and diabetes mellitus [12–14].

### Stroke diagnosis and severity

Stroke was diagnosed based on clinical presentation and neuroimaging using computerised tomography [12–14]. The severity of stroke symptoms at arrival was assessed by the National Institutes of Health for Stroke Scale (NIHSS) with a score range from no symptoms to severe stroke symptoms (NIHSS score = 0 to 42) [15].

### Clinical care quality

Care quality indicators were assessed using the standard SSNAP protocol [12] and outlined below. These indicators reflect the time-critical nature of acute stroke care including: neuroimaging; thrombolysis; swallow screening; reviews by a stroke specialist physician and nurse; and assessments by physiotherapy, occupational therapy, and speech and language therapy.

### Swallow screening

Swallow screening was conducted as soon as possible after stroke diagnosis and before patients had been given any oral fluid, food or medication. The following sequences of screening were performed by a trained healthcare professional for patients who had to be awake and alert for at least 15 min, in an upright position. Initially, the patient was given three spoons of water, and if there was no risk of aspiration, the patient was challenged with one cup of water. If successful, a trial was continued with a soft-diet meal [16].

### Nutrition status

The Malnutrition Universal Screening Test (MUST) protocol was used to identify patients at risk of malnutrition [16–18]. The information for MUST was completed by healthcare professionals prior to hospital discharge. This protocol is based on three independent variables: body mass index (BMI) score (BMI > 20 kg/m^2^ = 0, BMI 18.5–20 kg/m^2^ = 1, and BMI < 18.5 kg/m^2^ = 2), unplanned weight loss in the previous 3–6 months (weight loss < 5% = 0, weight loss 5–10% = 1, and weight loss > 10% = 2), and acute disease effect score (a score of 2 was added if a patient was recently affected by a disease and there was no nutrition intake or likely to be no nutrition intake for > 5 days).

### Disability

Pre-stroke and post-stroke disability was assessed by the modified Rankin Scale (mRS) prior to and also after the occurrence of stroke within the first 24 h of diagnosis and at discharge. Patients’ degree of disability or dependence on daily activities were based on mRS scores: 0 = no symptoms at all; 1 = no significant disability despite symptoms, able to carry out all usual duties and activities; 2 = slight disability, unable to carry out all previous activities but able to look after their own affairs without assistance; 3 = moderate disability; requiring some help, but able to walk without assistance; 4 = moderately severe disability, unable to walk without assistance and unable to attend to own bodily needs without assistance; 5 = severe disability, bedridden, incontinent and requiring constant nursing care and attention [19, 20].

### Nosocomial infections

Pneumonia and urinary tract infections (UTI) requiring antibiotic treatment acquired within 7 days of admission were documented [12–14].

### Thrombolysis and immediate thrombolysis-related complications

Thrombolysis, using the intravenous recombinant tissue plasminogen activator (rtPA) agent alteplase, was performed in patients who fulfilled criteria for therapy including confirmed diagnosis of acute ischaemic stroke (AIS), onset to arrival time of less than 3.5 h and without contra-indications [12–14]. Immediate thrombolysis-related complications (TRC) including severe hypertension, acute orolingual angioedema, anaphylaxis and hyperacute haemorrhage were defined clinically. Symptomatic intracranial haemorrhage (ICH) was identified by imaging evidence of intracerebral haemorrhage in conjunction with a significant decline in neurological function [12, 21].

### Level of care support planned at discharge

The planned level of care-support was documented, including: help for ADL, the frequency of home visits per week, and joint-care planning between health and social care for post-discharge management. Information was also documented on the decision to introduce palliative care by discharge date, as well as discharge to a new care home, either on a temporary or permanent basis [12–14].

### Categorisation of variables

Dichotomisation was applied for CHF, AF, previous stroke and hypertension, as well as in-patient infections according to the presence or absence of any history of the condition, and mortality. Moderately severe to severe disability at discharge was defined as an mRS score ≥ 4. Moderately severe to severe stroke on arrival was defined as an NIHSS score ≥ 16. Prolonged LOS on HASU was defined as those who stayed > 3 weeks (upper quartile). Swallow screening status was categorised into three groups: screening performed within 4 h, 4–72 h, and > 72 h of stroke diagnosis [12, 22]. A total sum of scores was used to categorize nutrition status: well-nourished (MUST score = 0) and at risk of malnutrition (MUST score ≥ 1) [23, 24].

### Statistical analysis

The associations of subject characteristics, management care and outcomes in relation to different location of stroke onset (HOS and COS) categories were explored by chi-squared tests. Multivariable logistic regression was conducted to estimate the risk of nosocomial infections within 7 days of admission; malnutrition; prolonged LOS on HASU; disability at discharge; in-patient mortality; and palliative care by discharge date (dependent variables) for patients with HOS — patients with COS were used as a reference group (independent variable). The results are presented as three models: *model 1*, unadjusted; mo*del* 2, adjusted for age, sex and co-morbidities; *model 3*, as in model 2 plus pre-stroke disability (mRS), type of stroke (ICH) and stroke severity (NIHSS), and expressed as odds ratios (OR) and 95% confidence intervals (CI). The goodness of fit for logistic regression was assessed by the Hosmer–Lemeshow test. Analyses were performed using SPSS Statistics for Windows, v.25.0 (IBM Corp., Armonk, NY, USA).

## Results

Stroke onset occurred with 272 (8.2%) in-hospital patients and 3037 in the community, with mean ages (± SD) of 80.2 years (± 12.5) and 76.4 years (± 13.5), and with equal sex distribution (47.1% men, 52.9% women: 50.3% men, 49.7% women), respectively. Compared to patients with COS, patients with HOS were on average older by 3.8 years (95%CI = 2.1–5.5, *P* < 0.001), with higher proportions ≥ 80 years of age (64.0% vs 46.4, *P* < 0.001); and with CHF (16.9% vs 4.9%, *P* < 0.001), AF (25.0% vs 19.7%, *P* = 0.036) and pre-stroke disability (9.6% vs 5.1%, *P* = 0.002). There were no group differences in a history of previous stroke, hypertension, diabetes, type of stroke, or severity of stroke at time of evaluation (Table1).

**Table 1:** Characteristics of patients with stroke onset in hospital compared to onset community

	Location of stroke onset		Group difference	
	Hospital (n = 272)	Community (n = 3037)	χ^2^	*P*
Sex (M: F)	47.1: 52.9	50.3: 49.7	1.1	0.304
Aged ≥ 80 years	62.0	46.4	24.6	** < 0.001**
Comorbidities				
Congestive heart failure	16.9	4.9	65.6	** < 0.001**
Atrial fibrillation	25.0	19.7	4.4	**0.036**
Stroke	24.3	23.0	0.2	0.649
Hypertension	53.3	52.2	0.1	0.716
Diabetes	17.3	15.9	0.3	0.563
Pre-stroke mRS (≥ 4)	9.6	5.1	9.6	**0.002**
Severity				
Intracranial haemorrhage	14.4	15.9	0.4	0.510
NIHSS at baseline (≥ 16)	7.0	6.8	0.1	0.932
Care quality				
Thrombolysis treatment of ischaemic stroke	8.8	17.1	10.3	**0.001**
Neuroimaging within 12 h	97.1	99.2	11.3	**0.001**
Admission to HASU within 72 h	43.8	97.8	1111	** < 0.001**
Swallow screen < 4 h	64.7	81.4	43.3	** < 0.001**
Swallow screen within 72 h	87.9	96.4	44.4	** < 0.001**
Stroke physician within 72 h	93.0	96.7	9.6	**0.002**
Stroke nurse within 72 h	95.2	98.5	14.6	** < 0.001**
Physiotherapy assessment within 72 h	83.5	90.5	12.4	** < 0.001**
Occupational therapy assessment within 72 h	77.9	87.7	12.7	** < 0.001**
Communication assessment by speech and language therapist within 72 h	51.1	48.5	0.7	0.405
Swallowing assessment by speech and language therapist within 72 h	41.2	35.8	3.1	0.077
Clinical outcomes				
Urinary tract infection within 7 days	8.6	7.6	0.4	0.536
Pneumonia within 7 days	19.6	10.5	19.3	** < 0.001**
Malnutrition by discharge	44.8	25.6	43.1	** < 0.001**
Immediate TRC	20.0	5.8	6.4	**0.011**
LOS on HASU > 3 weeks (top quartile)	44.9	23.5	40.6	** < 0.001**
Post-stroke mRS (≥ 4)	45.6	28.5	34.9	** < 0.001**
Death in hospital	24.6	13.6	25.5	** < 0.001**
Discharge support				
Palliative care	16.4	7.8	20.4	** < 0.001**
Care home discharge*	10.7	4.9	16.5	** < 0.001**
Help for activities of daily living	30.5	19.7	12.5	** < 0.001**
Joint care planning	30.9	22.6	9.5	**0.002**
Weekly visit	13.7	10.5	2.5	0.113

^*^Permanent and temporary care home discharge; *TRC*, thrombolysis-related complications; *LOS*, length of stay; *HASU*, hyperacute stroke units. Bold type indicates a statistical significant relationship

Table1 shows that compared to patients with COS, those with HOS had a lower proportion undergoing thrombolysis (for ischaemic stroke) or neuroimaging within 12 h. Within 72 h of evaluation, there were proportionally fewer HOS patients admitted to HASU, and who had swallow screening, as well as review by a stroke physician or nurse, or assessment by physiotherapy and occupational therapy. Both groups had similar communication and swallowing assessment by speech and language therapists. Amongst patients with HOS, 92.3% were eventually transferred to HASU.

**Fig. 1:**
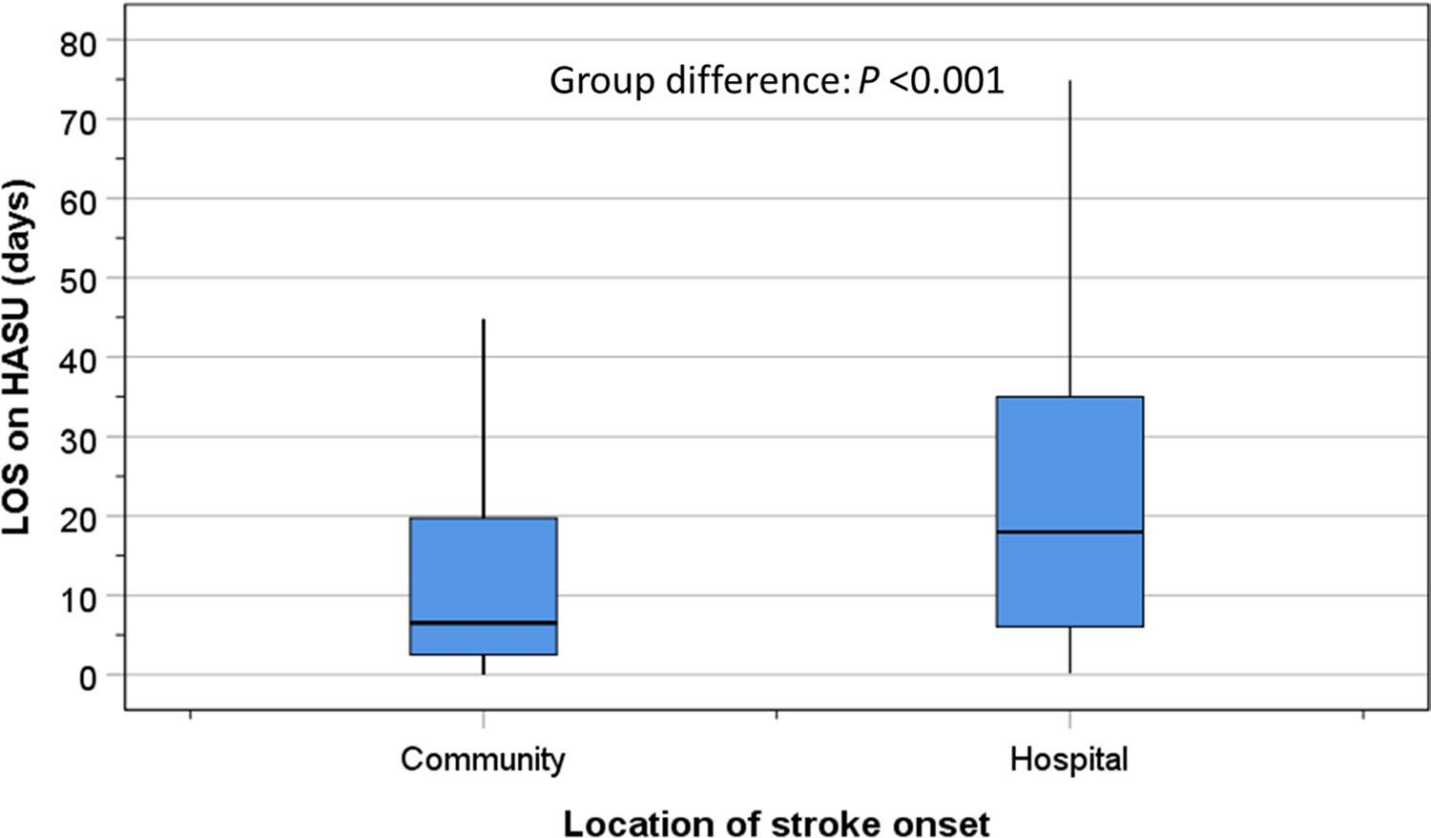
Box and whisker plot of length of stay on HASU amongst patient with stroke onset in the community compared with those with onset in hospital

Compared to patients with COS, the proportions of patients with HOS were greater with respect to: nosocomial pneumonia within 7 days of evaluation (19.6% vs 10.9%, *P* < 0.001); malnutrition (44.8% vs 25.6%, *P* < 0.001); LOS > 3 weeks (44.9% vs 23.5%, *P* < 0.001); disability at discharge (45.6% vs 23.5%, *P* < 0.001); and death in hospital (24.6% vs 13.6%, *P* < 0.001). There were no group differences in UTI (Table**1**). There was also increased care support needed by patients with HOS including: palliative care (16.4% vs 7.8%, *P* < 0.001); nursing home care (10.7% vs 4.9%, *P* < 0.001); help for ADL (30.5% vs 19.7%, *P* < 0.001); and joint-care planning (30.9% vs 22.6%, *P* = 0.002) (Table1). Amongst patients with HOS, the median LOS time spent in hospital before stroke onset was 3.2 days (IQR = 1.2–7.2 days). The median LOS on HASU for HOS patients (18.0 days; IQR = 6.0–35.0) was significantly (*P* < 0.001) longer than that for those with COS (6.5 days; IQR = 2.5–19.8) (Fig. 1).

### Comparison of risk of outcomes

**Fig. 2:**
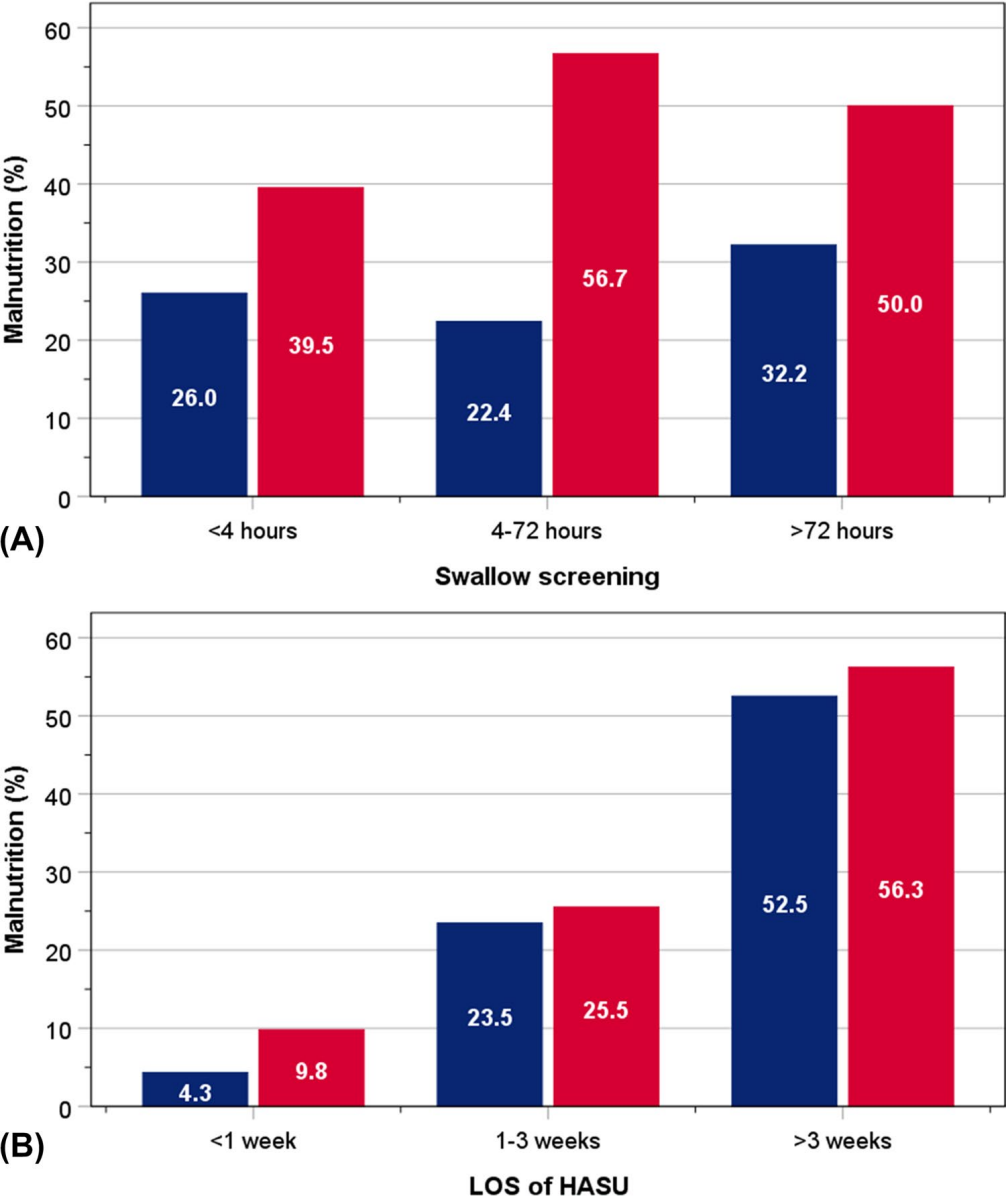
Proportions of patients with malnutrition in relation to time to swallow screening (**A**) length of stay on HASU (**B**) and amongst patient with stroke onset occurring in the community and in hospital

The risk of malnutrition was least common amongst patients who had swallow screening within 4 h (Fig. 2A) and for those who spent shorter LOS on HASU (Fig. 2B). This risk occurred more frequently amongst those who had swallow screening beyond 72 h and who spent the longest time on HASU. For any given time, the risk of malnutrition was consistently more common amongst patients with HOS.

After adjustments for age, sex and co-morbidities (CHF, AF, history of previous stroke, hypertension and diabetes), compared to patients with COS, patients with HOS were more likely to have pneumonia: OR = 1.9 (95%CI = 1.3–2.6); malnutrition: OR = 2.2 (95%CI = 1.7–2.9); thrombolysis complications: OR = 5.3 (95%CI = 1.5–18.2); LOS > 3 weeks: OR = 2.5 (95%CI = 1.8–3.4); post-stroke disability: OR = 1.8 (95%CI = 1.4–2.4); and in-hospital mortality: OR = 1.8 (95%CI = 1.2–2.4). Care support was also more likely to be needed including, palliative care: OR = 1.9 (95%CI = 1.3–2.8); nursing care home: OR = 2.0, 95%CI = 1.3–4.0, help for ADL: OR = 1.6 (95%CI = 1.1–2.2); and joint-care planning: OR = 1.5 (95%CI = 1.1–1.9). Further adjustments with pre-stroke disability, type and severity of stroke only marginally changed the associations between the location of stroke onset and outcomes (Table 2).

**Table 2:** Risk of adverse outcomes in hospital-onset stroke compared with community-onset stroke (reference group)

	Risk of adverse outcomes in hospital-onset stroke								
	Unadjusted			Adjusted for age, sex, and co-morbidities			Adjusted for age, sex, co-morbidities, prestroke mRS, ICH,** NIHSS**		
Clinical outcomes	OR	95% CI	*P*	OR	95% CI	*P*	OR	95% CI	*P*
Pneumonia	2.07	1.49–2.89	** < 0.001**	1.85	1.31–2.61	** < 0.001**	1.91	1.33–2.73	** < 0.001**
Malnutrition	2.36	1.81–3.06	** < 0.001**	2.20	1.68–2.89	** < 0.001**	2.29	1.73–3.23	** < 0.001**
Immediate TRC	4.06	1.26–13.05	**0.019**	5.27	1.51–18.42	**0.009**	5.17	1.47–18.17	**0.010**
LOS on HASU > 3 weeks (top quartile)	2.65	1.95–3.16	** < 0.001**	2.49	1.81–3.43	** < 0.001**	2.52	1.81–3.51	** < 0.001**
Post-stroke mRS (≥ 4)	2.10	1.64–2.71	** < 0.001**	1.81	1.39–2.36	** < 0.001**	1.93	1.44–2.58	** < 0.001**
Death in hospital	2.08	1.55–2.79	** < 0.001**	1.76	1.20–2.40	** < 0.001**	1.98	1.42–2.76	** < 0.001**
Discharge support									
Palliative care	2.32	1.59–3.37	** < 0.001**	1.88	1.27–2.79	**0.002**	2.02	1.34–3.05	**0.001**
Care home discharge	2.33	1.53–3.54	** < 0.001**	1.99	1.29–3.97	**0.002**	2.05	1.33–3.17	**0.001**
Help for activities of daily living	1.79	1.29–2.48	** < 0.001**	1.55	1.10–2.18	**0.013**	1.55	1.09–2.20	**0.015**
Joint care planning	1.53	1.17–2.00	**0.002**	1.47	1.11–1.93	**0.007**	1.42	1.07–1.88	**0.015**

*TRC*, thrombolysis-related complications; *LOS*, length of stay; *HASU*, hyperacute stroke units; co-morbidities: congestive heart failure, atrial fibrillation, hypertension, previous stroke, diabetes mellitus; *mRS*, modified Rankin Scale; *ICH*, intracranial haemorrhage; *NIHSS*, National Institutes of Health stroke severity. Care home discharge permanent and temporary.﻿ Bold type indicates a statistical significant relationship

## Discussion

### Summary

In this study of patients with HOS and COS, their characteristics including age and health status, management and outcomes have been described in detail. A number of additional factors were included that have seldom been addressed previously. All these, and the previously documented factors, are inextricably linked to outcomes of patients with HOS. Overall, our findings show that compared with COS patients, those with HOS were older, had greater pre-stroke disability and CHF or AF. There was delayed swallow screening, and a lower proportion met targets for timely neuroimaging, swallow screening, review by a stroke physician or nurse and physiotherapy and occupational therapy assessment. Consequently, HOS patients were at 1.5- to twofold greater risk of adverse outcomes including: pneumonia, malnutrition, TRC, post-stroke disability or mortality, and requirements for supportive care at discharge. Our findings support the need for greater attention on monitoring the progress of management and outcomes for this high-risk group of patients.

### Pre-stroke disability

Our observation of a greater prevalence of pre-stroke disability (mRS score ≥ 4) amongst HOS patients is novel. This index relates closely to several outcomes of stroke [20] including nosocomial infections, increased LOS on HASU, in-hospital mortality, help for ADL and nursing care [20, 25]. Thus, research increasingly demonstrates the ability of pre-stroke disability indices, and other similar tools such as the pre-fracture mobility index [20, 25, 26], to predict post-event outcomes.

### Age and co-morbidities

Patients with HOS were older and proportionally more had underlying health conditions, also been observed in previous studies, including CHF [6, 7] and AF [3, 6]. Arterial disease, which is the underlying aetiology of many cardiovascular diseases, has been more commonly identified in patients with HOS [4]. The proportions of hypertension and diabetes were present equally in both groups in this study, consistent with findings from previous studies [4]. In addition, a higher proportion of serious conditions such as active malignancies amongst patients with HOS has also been demonstrated [7, 27], which is likely to be due to cancer-associated hypercoagulation and migratory thromboembolism [28]. The observation of high proportions of patients undergoing surgical intervention with HOS [2, 4] may possibly be due to temporary discontinuation of antiplatelets or anticoagulants, especially those with a history of AF [29].

### Severity of stroke

Patients with HOS and those with COS in our study had similar proportions of severe stroke or ICH. This differed from some previous studies which reported greater severity with HOS patients [3, 6]. These factors did not therefore contribute towards differences in outcomes between HOS and COS in our study.

### Management

Several studies have indicated delays in recognition and assessment of patients with HOS [2, 8, 11], whilst others found no differences [4]. Our observation of a lower proportion of HOS receiving neuroimaging within 12 h was consistent with previous reports [7, 8]; and such patients were less likely to have a neurological examination [5]. Furthermore, evidence of a lower proportion of HOS patients undergoing swallow screening within the Royal College of Physicians target of 4 h of diagnosis is detrimental, as is the higher proportion with delayed swallow screening beyond 72 h. Delayed swallow screening is associated with poor outcomes in acute stroke patients [22], as early swallow screening is vital to allow correct decisions on nutritional support to be made, thus leading to better stroke recovery [30]. Coexisting acute conditions of patients with HOS and the complexities of hospital practice may be the underlying reasons for these differences [2]. These factors have all been shown to increase the incidence of several poor outcomes such as nosocomial pneumonia, prolonged LOS, disability and death [20, 22]. The observation that a lower proportion of HOS patients met targets for timely swallow screening, stroke physician or nurse review of physiotherapy and occupational therapy assessment provide further evidence for a lower standard of care quality received by this group of patients. There has been a report that patients with HOS received lower adherence to process-based quality measures (Get With the Guidelines Stroke achievement measures) [6], although the same group has reported opposite conclusions in an earlier paper [31].

### Clinical outcomes

A number of poorer clinical outcomes were identified amongst HOS patients in our study, extending previous observations of post-stroke disability [4, 5, 8] and mortality [3, 5–8]. This study also included risk of malnutrition, particularly amongst those with delayed swallow screening, prolonged LOS on HASU and TRC, which has not previously been well-documented. Malnutrition amongst patients admitted with an acute stroke is a significant indicator of post-stroke adverse outcomes including disability, mortality and prolonged LOS [16]. Few studies have also reported LOS; our observation of a median LOS on HASU of 18 and 6.5 days respectively for HOS and COS patients is very similar that of 17 and 8 days by Saltman et al. [8]. Malnutrition has profound effects on patient outcomes, particularly prolonged hospital LOS, which in itself, leads to many adverse outcomes including nosocomial infections and death, as well as sarcopenia due to the lack of mobility [32]. A holistic approach is therefore necessary to prevent malnutrition and its health consequences.

Although the proportion of patients selected for thrombolysis treatment amongst HOS was lower than that of COS patients, and similarly reported in other centres [4, 7, 8], immediate TRC were significantly greater amongst HOS patients. We are not aware of this finding in the existing literature, but it is important as TRC are associated with a four to five-fold increased risk of nosocomial infections, worsening stroke severity, longer HASU stay, disability at discharge and mortality, and palliation [21].

### Supportive care at discharge

We also observed that HOS patients required greater levels of supportive care at discharge, which has only rarely been documented, although some studies have reported that HOS patients are less likely to be discharged to their own home [5, 6, 8]. However, our findings of increased palliation, help with ADL and care-planning at discharge for patients with HOS have not been reported previously.

## Strengths and limitations

The present study consisted of a cohort of patients recruited consecutively from one of the largest NHS regions in the UK, with similar characteristics to the rest of the stroke population in the UK [12]. The proportion of patients with HOS of 8.2% is comparable with most major reports [1–5]. The data were examined using various logistic regression models to adjust for age, sex and co-morbidities, as well as additional adjustments for pre-stroke disability and stroke severity. All data were collected in accordance with the national SSNAP protocol which used standardised outcome measures including NIHSS for assessing stroke severity [15], mRS for assessing prestroke and poststroke disability [19], as well as with other measures commonly used in national stroke surveys such as nosocomial infections in the first 7 days of admission for acute stroke, [12] and nutritional screen and MUST protocol [17, 23, 24]. There were certain limitations to this study including the lack of information on post-discharge long-term outcomes such as readmission, disability and mortality. We did not conduct interventions on these patients; therefore, all treatment followed standard procedures. Future studies are suggested with more focus on treatment to see if outcomes could be improved among patients with HOS. In addition, specific primary conditions in patients admitted with HOS were not collected by SSNAP. However, our findings of higher proportions of individuals with AF and CHF, as well as older adults and pre-stroke moderately severe to severe disability (mRS score ≥ 4), indicate that most of the patients with HOS were initially admitted with conditions related to older age or cardiac complications.

In conclusion, we present a detailed analysis of underlying differences in subject characteristics between patients with HOS and those with COS and adverse consequences. Our findings provide further insights into the understanding of poorer outcomes associated with HOS, as well as evidence for clinicians and healthcare professionals to focus on quality improvement for patients with HOS. This group of patients, although relatively small, deserves to be monitored closely by national audit programmes.

## Data Availability

No additional data are available.

## Declarations

### Provenance and peer review

Not commissioned; externally peer reviewed.
